# Practical Notes on Diagnosis and Treatment

**Published:** 1910-07-30

**Authors:** 


### Practical Notes on Diagnosis and Treatment.

**Chorea.**

In most, if not all, cases of chorea the heart is dilated, a fact which is apt to be overlooked, but which is demonstrable by careful percussion. All cases should be treated by rest in bed, in addition to any drug treatment. Aspirin has produced better results than any other drug in common use, and appears to shorten the course of the disease.—*Dr. James Burnett.*

**Malignant Endocarditis.**

The changing character of the murmur, on which stress is commonly made, I have not found of great value, but the development of an aortic murmur in adult life should be regarded with the gravest suspicion. The aortic incompetence of later life, due to degenerative change can usually be readily differentiated. The fact that extensive vegetations may be present on the aortic valve without giving rise to a murmur, may render a correct diagnosis almost impossible.—*Sir John Broadbent.*

**Polio-encephalitis.**

As in polio-myelitis so in polio-encephalitis, the change which occurs after the onset of the disease is all towards improvement. The immunity given by one attack is usually complete, and relapses are extremely rare. The consequences most to be dreaded are mental degeneration and epilepsy. In mild cases where the cerebellum or mid-brain have been slightly affected and improvement has rapidly occurred, there is little likelihood of such sequelæ; but in the cortical cases, and in all instances in which there has been prolonged unconsciousness at the onset of the disease, both mental deterioration and epilepsy are prone to develop.—*Dr. R. Miller.*

**Dental Caries.**

Toothache connected with open carious teeth may be relieved by the local use of carbonate of sodium—½ drachm to the ounce of hot water.

**Burns.**

In superficial burns and scalds a saturated solution of bicarbonate of sodium applied on moistened cloth will quickly relieve the burning pain.

**Copper as an Emetic.**

Copper is a much better emetic for children than the time-honoured ipecacuanha, the action of which is very uncertain. The dose should be ¼ to ⅛ gr., repeated every ten minutes.—*Dr. Braine-Hartnell.*

**A Mouth Wash.**

Many diseases in aged persons arise from insanitary conditions of the mouth. A soft badger's hair tooth-brush should be recommended and a lotion, both astringent and antiseptic, should be prescribed, for example, Hazeline, glycerini boracis aa ℥ij, eau de Cologne, ℥j, aquam rosae ad ℥viij; one tablespoonful in a large wineglass of water as a mouth wash.

**Pyuria.**

In convalescence from pneumonia or typhoid fever there may be an amount of pus in the urine sufficient to cause anxiety, but as a rule the establishment of the normal health results in the prompt disappearance of the deposit. The same condition may occur in measles and diphtheria, and with rare exceptions should be regarded as a part of the elimination of the poisons of the disease analogous to the desquamation of the skin.—*Dr. J. H. Thursfield.*

